# Integrated UPLC-Q/TOF-MS Technique and MALDI-MS to Study of the Efficacy of YiXinshu Capsules Against Heart Failure in a Rat Model

**DOI:** 10.3389/fphar.2019.01474

**Published:** 2019-12-06

**Authors:** Jing Xu, Xianyu Li, Fangbo Zhang, Liying Tang, Junying Wei, Xiaoqing Lei, Huanhuan Wang, Yi Zhang, Defeng Li, Xuan Tang, Geng Li, Shihuan Tang, Hongwei Wu, Hongjun Yang

**Affiliations:** ^1^Institute of Chinese Materia Medica, China Academy of Chinese Medical Sciences, Beijing, China; ^2^Experimental Research Centre, China Academy of Chinese Medical Sciences, Beijing, China; ^3^Cardiovascular Center, China-Japan Friendship Hospital, Beijing, China

**Keywords:** heart failure, YiXinShu capsules, UPLC-Q/TOF-MS, MALDI-MS, metabonomic studies

## Abstract

**Background:** Yixinshu Capsules (YXSC) are widely used in Chinese medicine for the treatment of cardiovascular diseases. However, the therapeutic mechanisms of action are not well understood.

**Method:** In this study, a metabonomic approach based on integrated UPLC-Q/TOF-MS technique and MALDI-MS was utilized to explore potential metabolic biomarkers that may help increase the understanding of heart failure (HF) and in order to assess the potential mechanisms of YXSC against HF. Plasma metabolic profiles were analyzed by UPLC-Q/TOF-MS with complementary hydrophilic interaction chromatography and reversed-phase liquid chromatography. Moreover, time-course analysis at the 2nd, 4th, and 10th week after permanent occlusion was conducted. In an effort to identify a more reliable potential metabolic marker, common metabolic markers of the 2nd, 4th, and 10th week were selected through multivariate data analysis. Furthermore, MALDI-MS was applied to identify metabolic biomarkers in the blood at apoptotic positions of heart tissues.

**Results:** The results showed that HF appeared at the fourth week after permanent occlusion based on echocardiographic assessment. Clear separations were observed between the sham and model group by loading plots of orthogonal projection to latent structure discrimination analysis (OPLS-DA) at different time points after permanent occlusion. Potential markers of interest were extracted from the combining S-plots, variable importance for the projections values (VIP > 1), and t-test (*p* < 0.05). Twenty-one common metabolic markers over the course of the development and progression of HF after permanent occlusion were identified. These were determined to be mainly related to disturbances in fatty acids, phosphatidylcholine, bile acids, amino acid metabolism, and pyruvate metabolism. Of the metabolic markers, 16 metabolites such as palmitoleic acid, arachidonic acid, and lactic acid showed obvious changes (*p* < 0.05) and a tendency for returning to baseline values in YXSC-treated HF rats at the 10th week. Moreover, four biomarkers, including palmitoleic acid, palmitic acid, arachidonic, acid and lactic acid, were further validated at the apoptotic position of heart tissue using MALDI-MS, consistent to the variation trends in the plasma.

**Conclusions:** Taken in concert, our proposed strategy may contribute to the understanding of the complex pathogenesis of ischemia-induced HF and the potential mechanism of YXSC.

## Introduction

Heart failure (HF), a common clinical syndrome, is commonly associated with high morbidity and, if survived, may result in lifelong and often devastating health conditions (Doenst et al., 2013; Jackson et al., 2015; Ponikowski et al., 2016). As the end stage of various heart diseases, HF manifestation is characterized with architectural changes or functional disorders, including changes in electrophysiology, inflammatory responses, oxidative stress, energy metabolism, etc. (Doenst et al., 2013; Tanai and Frantz, 2015). Even though modern state-of-the art treatment options may be used to improve clinical symptoms and slow the progression of contractile dysfunction (Ohn and Ognoni, 2001; Pfeffer, 2003; Savarese et al., 2013), no significant breakthroughs have been made to improve the overall outcome of HF patients, indicating that HF prognosis and treatment remain poorly controlled (Guo et al., 2014; Yang et al., 2015).

Traditional Chinese medicine (TCM) has long been practiced using multiple components to treat as well as prevent various complex and refractory disease states. Yixinshu Capsule (YXSC), recorded in the Chinese Pharmacopoeia (Chinese Pharmacopoeia Commission, 2015), has been used for the treatment of HF in order to improve clinical symptoms such as chest pain, palpitation, shortness of breath, and cyanosis (Carubelli et al., 2015; Sun et al., 2017). YXSC, derived from a classic TCM prescription named Sheng-Mai-San, contains seven Chinese herbal medicines including *Salvia miltiorrhiza* Bunge, radix and rhizome; *Panax ginseng* C.A.Mey., radix and rhizome; *Ophiopogon japonicus* (Thunb.) Ker Gawl, radix; *Crataegus pinnatifida* Bunge, fructus; *Astragalus membranaceus* (Fisch.) Bunge, radix.; *Ligusticum chuanxiong* S.H.Qiu, Y.Q.Zeng, K.Y.Pan, Y.C.Tang & J.M.Xu, rhizome; *Schisandra chinensis* (Turcz.) Baill., fructus. The ratios of the above botanical materials in the preparation were 2:1.5:1.5:1.5:1.5:1:1. In our previous studies, a total of 276 components in the YXSC were identified mainly including ginsenosides, astragalus saponins, lignans, phenolic acids, and tanshinones (Wang et al., 2015). The quality control and evaluation of YXSC in this study can be seen in the **Supplementary Materials**. Moreover, previously conducted studies have shown that YXSC, as a standardized product, has been able to reduce mitochondrial-mediated apoptosis (Zhao et al., 2016), oxidative stress injury (Zhang et al., 2017), and myocardial dysfunction (Zhang et al., 2017). Even though numerous clinical trials have shown that YXSC exhibits a protective function against HF, the mechanism of YXSC used as a treatment option for HF remains unclear.

HF as a metabolic syndrome has been shown to be accompanied by metabolic derangements such as systemic and myocardial insulin resistance, mitochondrial dysfunction, and myocardial energetic failure associated with biosynthesis and metabolism of ATP, glycolysis, TCA cycle, and related metabolic pathways (Stanley et al., 2005). There is growing evidence to support the concept that alterations in substrate metabolism of HF significantly contribute to contractile dysfunction and the progression of LV remodeling (Birkenfeld et al., 2018). Therefore, metabolomics offers a suitable way to understand disease pathogenesis and the mechanisms of YXSC from the standpoint of a metabolic evaluation. Furthermore, some specific molecular targets (e.g., fatty acid–binding protein 3 and cytoskeleton-associated protein 5), regulated by YXSC against HF, were identified by proteomic analysis in a study reported by us previously (Wei et al., 2019). However, the metabolic phenotype of HF as well as the metabolic regulation of YXSC against HF remains incomplete and therefore unclear.

In this study, an untargeted metabolic profile approach based on UPLC-Q/TOF-MS with complementary hydrophilic interaction chromatography (HILIC) as well as reversed-phase liquid chromatography was used to investigate the metabolic changes of plasma in rats subjected to ischemia-induced HF. In an effort to identify more reliable potential metabolic biomarkers, common metabolic markers of different time points after myocardial infarction (MI) were selected through multivariate data analysis. These potential metabolic markers related to the perturbed metabolic pathways in HF rats were identified in order to develop an improved understanding of HF pathogenesis as well as the underlying mechanisms of YXSC. Moreover, MALDI-MS was further applied to track the identified metabolic biomarkers in the blood at the apoptotic position of the heart tissue. Compared with conventional analytical techniques, MALDI-MSI has the ability of *in situ* localization of a wide range of biomolecules in a simultaneous fashion from a tissue specimen in one single run. As a consequence, MALDI-MSI has become one of the most powerful techniques in the field of biomedical research and exhibits further applicability in disease diagnosis and prognosis, biomarker discovery, and drug development (Wang et al., 2015). Hopefully, this study will provide a useful approach for exploring the mechanism of ischemia-induced HF and evaluating the efficacy of YXSC in regards to the study of metabolites associated with HF.

## Materials and Methods

### Animals, Chemicals, and Reagents

A total of 28 adult male Sprague-Dawley rats (body weight: 260–270 g) were provided by the Animal Breeding Centre of Beijing Vital River Laboratories Company (Beijing, China). All experimental animal procedures were approved by the Academy of Chinese Medical Science’s Administrative Panel on Laboratory Animal Care and performed in accordance with institutional guidelines and ethics of the committee as part of the China Academy of Chinese Medical Sciences (February 1st, 2016).

YXSC was obtained from Guizhou Xinbang Pharmaceutical Co., Ltd (Guizhou, China); Valsartan was purchased from HaiNan Aumei Pharmaceutical Co., Ltd (Hainan, China). All organic solvents used throughout the studies were of HPLC-grade and were purchased from Fisher Scientific (Shanghai, China). All other chemicals used were of analytical grade and purchased from Sigma-Aldrich (Shanghai, China) unless stated otherwise.

### Animal Models, Drugs Treatment, and Echocardiography

Male Sprague-Dawley rats were kept inside an animal room at a temperature of 22 ± 2°C and a relative humidity of 50 ± 10%, with a 12 h light/12 h dark cycle. All animals were acclimatized for 1 week prior to surgery. The animals had free access to water and fodder (Beijing Keaoxieli Co, Ltd.). A total of 28 rats were randomly divided into sham rats (n = 8) and rats with MI (n = 20). According to previously published methods, ischemia-induced HF was performed by ligation of the left anterior descending coronary artery near its origin from the left coronary artery (Huang et al., 2006; Turer, 2013). Rats in the sham group were subjected to a similar procedure except for the left coronary artery ligation. At the fourth week after permanent occlusion, HF appeared based on the value of ejection fraction (EF). Then, rats with MI were randomly divided into two groups of model group and YXSC group. Meanwhile, the YXSC group was orally administered YXSC (0.32 g· kg^-1^· day^-1^) equivalent to clinical doses for six consecutive weeks. Before occlusion and at the 4th and 10th week after occlusion, 2-D echocardiography (Visual Sonics, Canada) was employed to measure echocardiographic parameters; the left ventricular end-diastolic volume (LVEDV) and left ventricular end-systolic volume (LVESV) were calculated. Then, the values of EF of the LV were calculated from the LV dimensions as follows: EF (%) = (LVEDV - LVESV)/LVEDV × 100% (Zhang et al., 2014). Of the 20 rats with MI, 4 rats with MI died after surgery. Moreover, one rat in model group died at the 4th week and one rat died in sham group at the 10th week after occlusion.

### Sample Collection and Preparation

*Preparation of plasma samples*. In order to investigate the metabolic changes during the period from occlusion to HF, blood plasma samples were collected from the ophthalmic vein at the 2nd, 4th, and 10th week after occlusion. After centrifugation at 3,500 rpm for 10 min at 4 °C, the plasma was collected and stored at – 80 °C prior to metabonomic profiling analysis. Before UPLC-Q/TOF–MS analysis, 100 µl plasma was mixed with 300 µl of cold acetonitrile and then vortexed vigorously for 30 s. The resulting mixture was then centrifuged at 4 °C at 12,000 rpm for 10 min and the supernatant was injected into the UPLC-Q/TOF–MS analytical system.

*Tissue sectioning*. At the 10th week after occlusion, Sprague−Dawley rats were euthanized with pentobarbital (0.55 mg/kg, i.p.) and the heart tissues were explanted. Left and right atria were resected respecting anatomic landmarks. Then heart tissue was divided into two parts along the long axis section of left ventricle and snap-frozen in liquid nitrogen for further study. The slices of all tissues samples were sectioned at 10 µm thickness using a Leica CM1950 cryostat (Leica Microsystems GmbH, Wetzlar, Germany) at −17 °C and thaw mounted onto indium tin oxide (ITO) coated glass slides. The glass slides were then placed into a vacuum desiccator for approximately 1 h before matrix application. For MALDI-MS, the matrix solution, i.e., 1,5-DAN hydrochloride in 50% ethanol/water prepared as reported in the literature (Liu et al., 2014), was sprayed onto the tissue sections mounted onto ITO coated glass slides using an automatic matrix sprayer (ImagePrep, Bruker Daltonics). During this procedure, homogeneous matrix coverage was ensured over the entire tissue surface. For locating the apoptosis site of the left ventricle of the heart section, a TUNEL assay (11684795910, Roche) was performed according to the manufacturer’s specifications. Briefly, after dewaxing and hydrating, the parallel sections were treated with protease-K and freshly prepared TdT enzyme in a dark place, followed by sealing using the sealing ﬂuid, color development with DAB. Images were obtained with a DP72 digital camera (Olympus, Tokyo, Japan).

### Untargeted Metabolic Profiles Analysis by UPLC-Q/TOF-MS

An untargeted metabonomics approach based on complementary HILIC and reversed-phase liquid chromatography (RPLC) combined with Q-TOF mass spectrometry was implemented in order to select more potential biomarkers in the plasma of HF rats.

For HILIC-MS analysis, the separation was performed using a Waters UPLC BEH amide column (2.1 mm × 100 mm, 1.7 µm particle size). The mobile phase consisted of solvent A (0.1% formic acid–acetonitrile, containing 1 mM ammonium formate) and solvent B (0.1% formic acid–water, containing 2 mM ammonium formate). Gradient elution was employed (0–1 min, 95% A; 1–9 min, 95–50% A; 9.1–13 min, 50–95% A). The flow rate of the mobile phase was 0.3 ml min^-1^, the column temperature was 40 °C, and the injection volume was 1 µl. For RPLC-MS analysis, the separation procedure was carried out using s Waters ACQUITY HSS T3 (2.1 mm × 100 mm, 1.8 µm) system. The mobile phase consisted of solvent A (0.1% formic acid–water) and solvent B (0.1% formic acid–methanol). Gradient elution was employed (0–1 min, 100% A; 1–4 min, 100–30% A; 4–12 min, 30–0% A; 12.1–14 min, 0–100% A). The flow rate of the mobile phase was 0.3 ml min^-1^ and the injection volume was 1 µl.

The conditions of MS analysis were as follows: RPLC-MS and HILIC-MS spectra were acquired on a hybrid quadrupole-time-of-flight (Q-TOF) mass spectrometer (Xevo G2 Q-TOF MS systems, Waters Corp., Milford, MA, USA), equipped with an electrospray ionization (ESI) source. The full-scan data were acquired from 50 to 1,200 Da, with a scan time of 0.2 s using a capillary voltage of 3.0 kV for positive mode and 2.2 kV for negative mode, a desolvation temperature of 350 °C, sample cone voltage of 40 V, extraction cone voltage of 4 V, source temperature of 100 °C, cone gas flow of 40 L/h, and desolvation gas flow of 800 L/h. The mass spectrometer was calibrated across a mass range of 50–1,200 Da using a solution of sodium formate. The mass was corrected during acquisition using an external reference (Lock-Spray™) consisting of a 0.2 ng ml^-1^ solution of leucine enkephalin, infused at a flow rate of 5 µl min^-1^ *via* a lock spray interface and generating a reference ion at 556.2771 Da ([M+H]^+^) or 554.2615 Da ([M-H]^-^). The lock spray scan time was set to 0.5 s, with an interval of 15 s, and the acquired data were averaged over three scans. RPLC-MS spectra were obtained in positive and negative ion modes, respectively. For HILIC-MS analysis, only positive mode was employed. The system was controlled by the software package Masslynx V4.1.

### Matrix Assisted Laser Desorption Ionization Mass Spectrometry

MALDI-MS experiments were performed on an Ultraflextreme MALDI-TOF/TOF MS (Bruker Daltonics, Billerica, MA) equipped with a smartbeam Nd: YAG 355 nm laser operating at 2,000 kHz. Medium focus was set for the laser spot size (laser spot diameter: ∼50 µm); the laser power was optimized prior to each run and then fixed during the entire experiment. The mass spectra were acquired in negative reflectron mode, with a pulsed ion extraction time of 130 ns, an accelerating voltage of 20.00 kV, an extraction voltage of 17.95 kV, a lens voltage of 7.5 kV, and a reflector voltage of 21.10 kV. The mass spectra data were acquired at a mass range of m/z = 50–1,360 Da. The imaging data for each array position consisted of 200 laser shots, with spatial resolution of 200 µm for all heart tissue sections. External mass calibration was performed with standards before data acquisition. MALDI mass spectra were normalized with the total ion current (TIC). The signal intensity of each imaging data displayed was the normalized intensity. MS/MS fragmentations observed on the MALDI-TOF/TOF MS instrument in the LIFTTM mode were used for further detailed structural confirmation of the identified metabolites (Liu et al., 2014).

### Data Processing and Statistical Analysis

UPLC-Q/TOF-MS data processing was performed using the Progenesis QI software, which was developed for processing metabolic profiling data. Raw data obtained from the Masslynx 4.1 workstation of UPLC-Q/TOF-MS were imported to Progenesis QI. The detailed workflow included retention time correction, experimental design setup, peak picking, normalization, deconvolution, and alignment. Retention time (t_R_)-m/z datasets were used to characterize the detected ions. The rule of a coefficient of variation (CV) less than 30% was used to filter the consistent ions. The intensity of each t_R_-m/z variable was normalized and further imported into the SIMCA-P 13.0 software package for orthogonal partial least squares discriminant analysis (OPLS-DA). The OPLS-DA data were then used to identify the different metabolites responsible for the separation between the groups.

For MALDI-MS, regions of interest (ROIs) were located at the apoptotic area according to TUNEL staining results and the corresponding average intensity of metabolites was acquired. A two-tailed Student’s t-test was performed to compare the average intensity of metabolites between the different regions of the heart tissue. *p*-values ≤ 0.05 were considered statistically significant.

All other values measured *in vivo* were presented as means ± standard error. Statistical significance was determined *via* a one-way ANOVA test, followed by Tukey multiple comparison test or Student’s *t*-tests. A value of *p* ≤ 0.05 was considered statistically significant.

## Results and Discussion

### Echocardiographic Assessment

**Figure 1:**
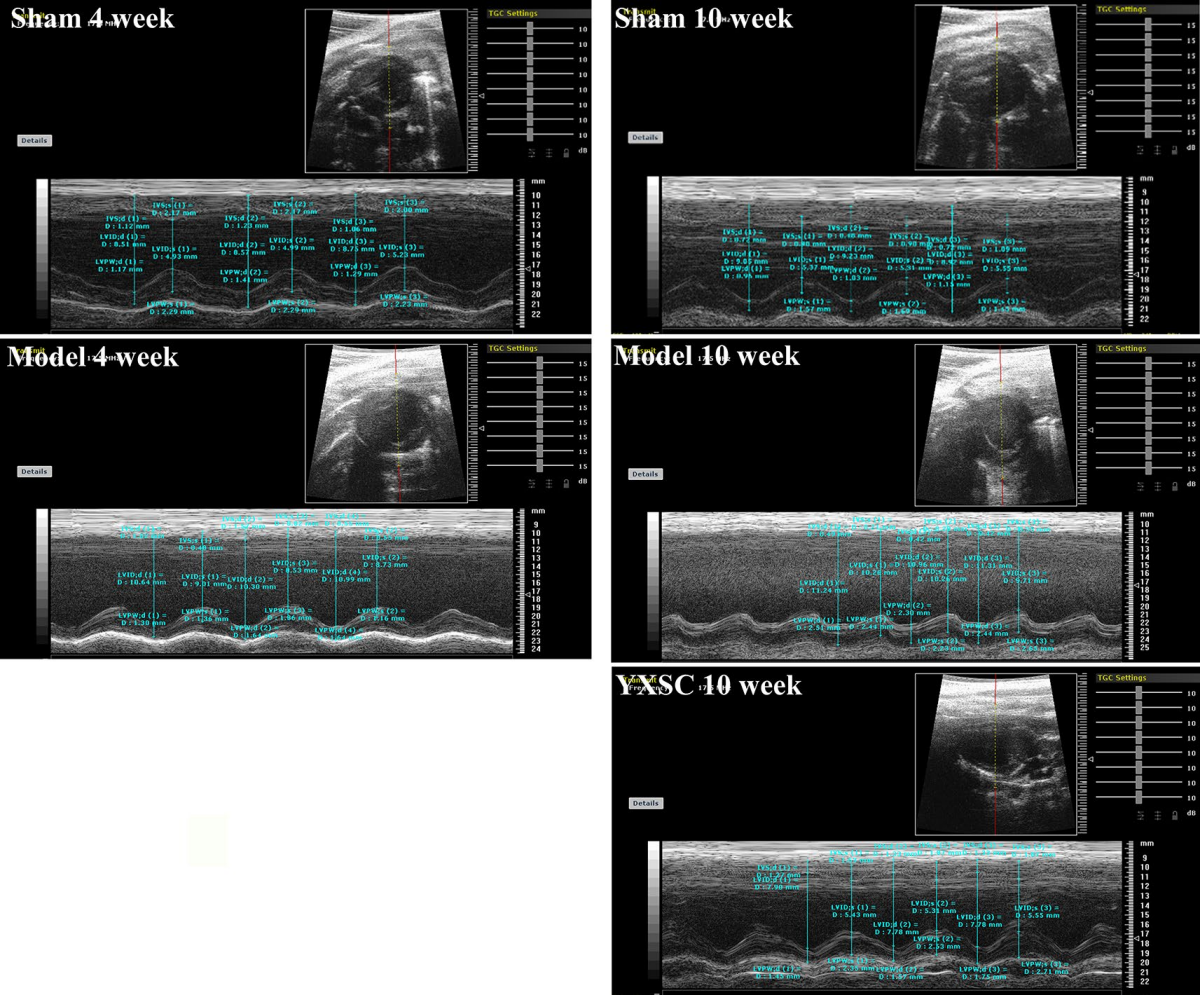
Representative echocardiographic in sham group, model group and YXSC group.

HF generally describes a final syndrome before mortality, triggered by a variety of factors. Decreased EF has already been recognized as a prominent standard in the diagnosis of HF and guiding HF management (Fu et al., 2015; Bloom et al., 2017). In our previous study (Wei et al., 2019), the protective effect of varying doses of YXSC on cardiac performance was evaluated by dynamic changes of EF. Here the dose of 0.32 g· kg^-1^· day^-1^ exhibited a beneficial effect and was therefore selected for further studies. As shown in **Figure 1** and **Table 1**, rats in model group significantly decreased in fractional shortening (FS) and EF at the 4th and 10th week after permanent occlusion compared to those of the sham group (*p* < 0.01). The results indicated heart dysfunction occurred. At the 10th week after myocardial ischemia, the YXSC group exhibited a reversed increase in FS and EF compared to the model group (*p* < 0.05), indicating that administration of YXSC had an effective cardio-protective effect against HF.

**Table 1:** Echocardiographic parameters of rats at 4th and 10th week.

	4th week		10th week		
	Sham	Model	Sham	Model	YXSC
LVEDd (mm)	8.05 ± 0.31	9.72 ± 1.22^**^	8.71 ± 0.30	10.15 ± 1.41^**^	9.13 ± 0.65^#^
LVEDs (mm)	5.24 ± 0.29	7.68 ± 1.27^**^	5.15 ± 0.27	7.98 ± 0.98^**^	6.26 ± 0.81^#^
EF (%)	69.00 ± 1.58	42.18 ± 5.74^**^	69.24 ± 1.56	41.49 ± 10.28^**^	54.89 ± 5.15^#^
FS (%)	43.32 ± 1.64	21.35 ± 3.25^**^	40.61 ± 1.25	21.75 ± 6.11^**^	32.28 ± 5.96^##^

LVEDd: left ventricle end-diastolic diameter; LVEDs: left ventricle end-systolic diameter; EF: ejection fraction; FS: fractional shortening; *p < 0.05, **p < 0.01, model group vs. sham group; ^#^p < 0.05, ^##^p < 0.01, YXSC group vs. model group.

### Plasma Untargeted Metabolic Profiles Analysis

**Figure 2:**
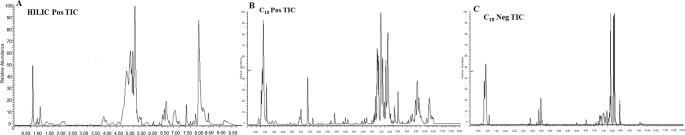
Typical TIC of the rat plasma obtained in ESI positive and negative mode based on HILIC-MS and RPLC-MS. **(A)** HILIC-MS TIC in positive mode; **(B)** RPLC-MS TIC in positive mode; **(C)** RPLC-MS TIC in negative mode.

HF is not an independent disease, but a progressive clinical syndrome and, unfortunately, represents the end stage of heart disease development. In this study reported herein, the obvious appearance of HF induced by ischemia occurred at the fourth week after permanent occlusion. In an effort to shed further light on metabolic profiling, various plasma metabolites were analyzed dynamically at the 2nd, 4th, and 10th week after permanent occlusion. The identification of common metabolic markers between sham and model groups at different time points may provide a dynamic view of the underlying pathological process of HF. Different types of mobile phases, including 0.05% and 0.1% formic and acetic acid in water, acetonitrile, and methanol were screened for plasma sample analysis. Optimized conditions of mobile phases for HILIC-MS and RPLC-MS were obtained as detailed in the section *Untargeted Metabolic Profiles Analysis by UPLC-Q/TOF-MS*. Typical total ion chromatograms (TICs) of plasma samples for HILIC-MS in the positive ionization mode as well as RPLC-MS chromatograms in the positive and negative ionization modes are shown in **Figure 2**.

QC samples were used to monitor the stability of the UPLC-Q/TOF-MS system. The QC samples were analyzed in the positive and negative ionization mode at regular intervals (every 10 samples) throughout the entire sequence. The RSDs of the peak areas and retention times of the detected ion signals were calculated. More than 80% of the RSDs were less than 30% for all QC samples in the negative and positive ionizations modes for all run sequences. Therefore, both the repeatability and stability of the global experimental performances were high and acceptable.

### Statistical Analysis and Identification of Potential Biomarkers

After deconvolution, alignment, and normalization using the Progenesis QI software, all ion signals of HILIC-MS and RPLC-MS chromatograms both in positive and negative ionization modes were extracted and merged in an excel file. Then the merged data were imported into the SIMCA-P software for multivariate statistical analysis. In order to eliminate any non-specific effects of the operative technique and to uncover the metabolic biomarkers, OPLS-DA was applied to compare metabolic changes in the model group with the sham group. As a supervised pattern recognition technology, OPLS-DA can be used to improve biomarker discovery efforts and separate samples into two blocks. This technique can then be used to improve discrimination between the two groups. The data were standardized using a Pareto-scaling technique for OPLS-DA.

**Figure 3:**
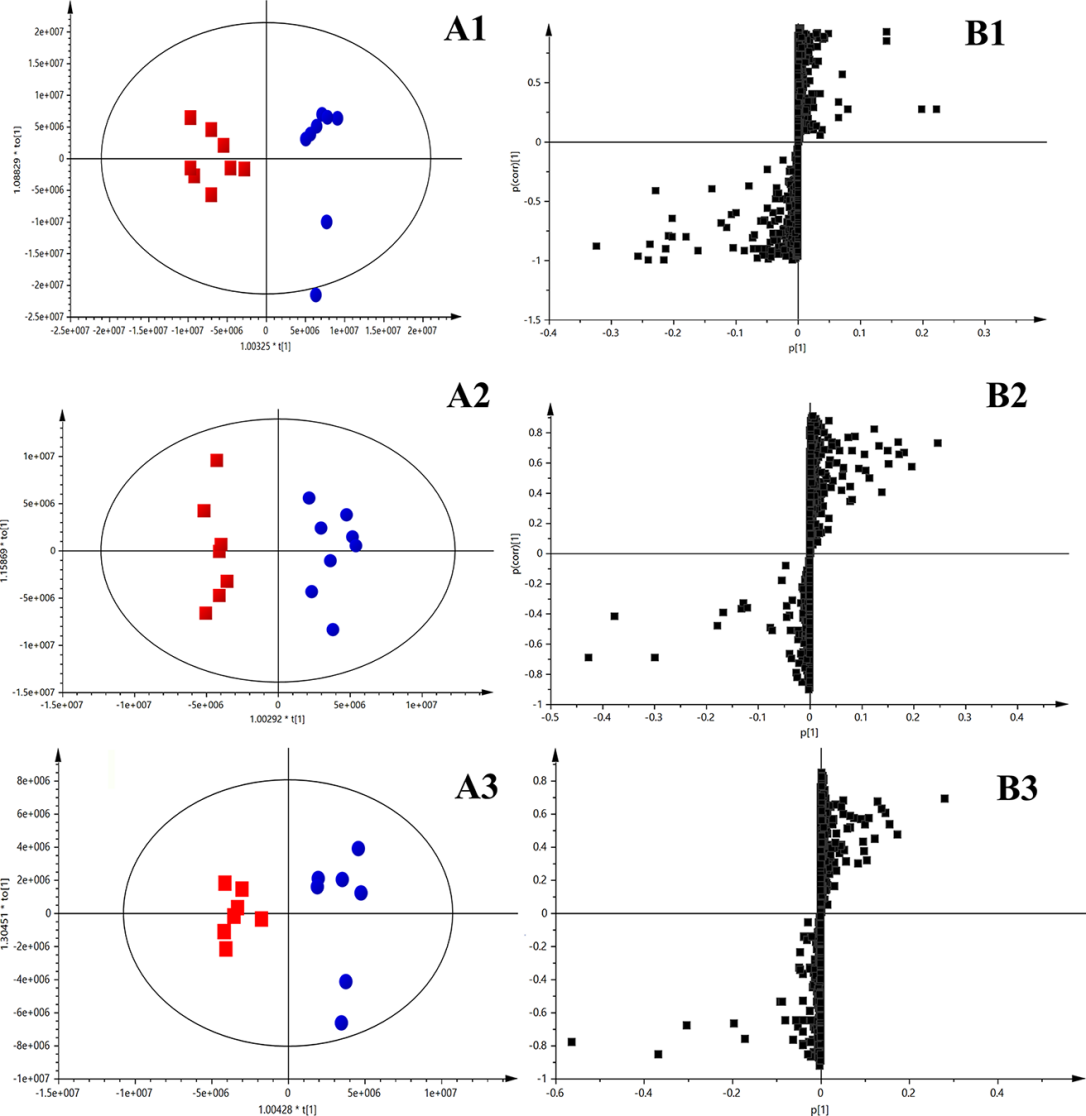
OPLS-DA results between the sham group and model group based on the merged data of plasma untargeted metabolic profiles. **(A)** OPLS-DA scores plot, sham group (blue) vs. model group (red); **(A1)**, 2nd week after occlusion; **(A2)**, 4th week after occlusion; **(A3)**, 10th week after occlusion; **(B)** S-plot constructed from OPLS-DA; **(B1)**, 2nd week; **(B2)**, 4th week; **(B3)**, 10th week. The ions (black square) furthest away from the origin contribute significantly to be responsible for the separation between sham and model group.

Score plots from OPLS-DA (**Figures 3 A1–A3**) indicated an obvious separation between the sham group and the corresponding model group of the 2nd, 4th, and 10th week after permanent occlusion, suggesting that blood metabolic perturbation significantly occurred in the model groups after permanent occlusion. From the corresponding S plots (**Figures 3B1–B3**), the ions (the dots in the figure) furthest away from the origin contributed significantly to be responsible for the separation between sham and model group and may be therefore regarded as the differentiating metabolites for HF. After combining the results of the S plots, the VIP value (VIP > 1) obtained from the OPLS-DA analysis, and the *t*-test (*p* < 0.05), the corresponding metabolites could be selected. Finally, common metabolite markers between the sham group and model group of the 2nd, 4th, and 10th week after permanent occlusion were selected as listed in **Table 2**. The chemical structures of the metabolites were identified according to online databases of the Human Metabolome Database (www.hmdb.ca), Metlin (https://metlin.scripps.edu) and the Mass Bank (www.massbank.jp) vWhen necessary, further confirmation was obtained through comparisons with authentic standards, including retention times and MS/MS fragmentation patterns.

**Table 2:** Identified common metabolic markers between the sham group and model group of the 2nd, 4th, and 10th week after permanent occlusion.

No.	Column	Name	Retention time(min)	Mean measured mass (Da)	Elemental composition	Normalized content (×10000) at the tenth week after MI		
						Sham	Model	YXSC
1	Waters UPLC HSS T3 column	PC(20:4/18:2)	9.34	806.5690 [M+H]^+^	C_46_H_80_NO_8_P	440.37 ± 14.20	348.28 ± 30.85↓^***^	474.34 ± 36.61↑^###^
2		PC(20:4/20:4)	9.26	830.5687 [M+H]^+^	C_48_H_80_NO_8_P	346.10 ± 34.55	307.64 ± 92.14↓^^^	304.21 ± 108.18
3		PC(18:2/16:0)	9.72	758.5695 [M+H]^+^	C_42_H_80_NO_8_P	1548.02 ± 89.64	1282.69 ± 95.20↓^**^	1662.92 ± 70.04↑^###^
4		PC(18:0/18:2)	10.45	786.6009 [M+H]^+^	C_44_H_84_NO_8_P	727.59 ± 104.42	540.20 ± 108.59↓^*^	711.10 ± 76.11↑^#^
5		PC(20:4/18:0)	10.32	810.6000 [M+H]^+^	C_46_H_84_NO_8_P	1910.97 ± 207.55	1617.89 ± 163.09↓^*^	1656.79 ± 731.39↑
6		PC(22:6/18:0)	10.17	834.5979 [M+H]^+^	C_48_H_84_NO_8_P	482.14 ± 70.77	405.19 ± 52.18↓^^^	487.59 ± 40.83↑^#^
7		3-Methyl-2-oxovaleric acid	3.92	129.0554 [M-H]^-^	C_6_H_10_O_3_	67.33 ± 14.16	82.67 ± 16.09↑^^^	67.71 ± 11.70↓
8		Palmitoleic acid	7.85	253.2168 [M-H]^-^	C_16_H_30_O_2_	55.05 ± 9.61	82.82 ± 4.61↑^***^	69.71 ± 11.39↓^#^
9		Palmitic acid	8.13	255.2326 [M-H]^-^	C_16_H_32_O_2_	626.17 ± 137.94	689.71 ± 77.81↑^^^	594.43 ± 30.87↓^#^
10		Chenodeoxycholic acid	6.48	391.2847 [M-H]^-^	C_24_H_40_O_4_	24.34 ± 18.57	11.29 ± 5.38↓^^^	33.56 ± 16.49↑^#^
11		Taurochenodesoxycholic acid	6.64	498.2889 [M-H]^-^	C_26_H_45_NO_6_S	5.53 ± 0.49	3.35 ± 1.04↓^**^	4.31 ± 2.94↑
12		Arachidonic acid	7.94	303.2325 [M-H]^-^	C_20_H_32_O_2_	223.80 ± 30.08	277.56 ± 16.26↑^**^	234.89 ± 16.79↓^##^
13		Taurocholic acid	6.07	514.2837 [M-H]^-^	C_26_H_45_NO_7_S	49.41 ± 3.43	20.52 ± 1.37↓^***^	34.18 ± 22.37↑
14		Lactic acid	1.11	89.0243 [M-H]^-^	C_3_H_6_O_3_	15.16 ± 4.51	29.15 ± 4.42↑^**^	19.41 ± 1.54↓^##^
15		Citric acid	1.10	191.0193 [M-H]^-^	C_6_H_8_O_7_	97.99 ± 15.72	74.35 ± 10.84↓^*^	90.27 ± 5.78↑^#^
16		Uric acid	1.11	167.0206 [M-H]^-^	C_5_H_4_N_4_O_3_	16.14 ± 1.86	28.49 ± 3.36↑^***^	21.17 ± 1.63↓^##^
17	Waters UPLC BEH Amide column	Valine	6.73	118.086255[M+H]^+^	C_5_H_11_NO_2_	88.77 ± 25.20	39.26 ± 65.22↓^*^	87.05 ± 27.22↑^#^
18		Edetic acid	8.11	293.098824[M+H]^+^	C_10_H_16_N_2_O_8_	171.64 ± 21.51	149.48 ± 160.70↓^^^	277.37 ± 20.52↑^###^
19		Creatine	7.57	132.076753[M+H]^+^	C4H9N3O2	34.71 ± 4.09	23.09 ± 31.67↓^**^	39.26 ± 9.12↑^##^
20		L-acetylcarnitine	5.10	204.123034[M+H]^+^	C_9_H_17_NO_4_	161.58 ± 34.87	148.53 ± 154.90↓^^^	250.98 ± 66.68↑^##^
21		N1-Methyl-2-pyridone-5-carboxamide	8.52	170.093359[M+NH_4_]^+^	C_7_H_8_N_2_O_2_	12.85 ± 2.01	10.37 ± 12.49↓^^^	16.67 ± 2.85↑^##^

* p < 0.05, ** p < 0.0, *** p < 0.001 and ^^^ VIP > 1; model group vs. sham group.

^#^p < 0.05, ## p < 0.01, ^###^p < 0.001; YXSC group vs. model group.

As shown in **Table 2**, detail information on the filtered common metabolic markers is listed, including the applied chromatographic columns, names, retention times, measured mass (m/z), and the relative contents at the 10th week after permanent occlusion. Of the total 21 metabolic markers, 6 PCs, 3 fatty acids, 3 bile acids, 2 amino acids, and 7 other basic metabolites including lactic acid, citric acid, uric acid, etc., could be identified.

The levels of six PCs [PC (20:4/18:2), PC (20:4/20:4), PC (18:2/16:0), PC (18:0/18:2), PC (20:4/18:0), PC(22:6/18:0)], three bile acids (chenodeoxycholic acid, taurocholic acid and taurochenodesoxycholic acid), two amino acids (valine and creatine), and four other basic metabolites (edetic acid, citric acid, L-acetylcarnitine and N1-Methyl-2-pyridone-5-carboxamide) obviously decreased in the model group compared to the sham group (*p* < 0.05 or VIP > 1). However, the levels of three fatty acid species (palmitoleic acid, palmitic acid, and arachidonic acid) and three other basic metabolites (lactic acid, uric acid, and 3-methyl-2-oxovaleric acid) showed incremental changes in the model group compared to the sham group (*p* < 0.05 or VIP > 1).

After administration of YXSC, among the total of 21 metabolic markers, the levels of 16 metabolites (4 PCs, 3 fatty acids, 2 amino acids, 1 bile acids and 6 other metabolites) showed significant changes in the YXSC group compared to the model group and were found to return to normal levels as listed in **Table 2**.

### *In Situ* Mass Spectrometry Imaging

**Figure 4:**
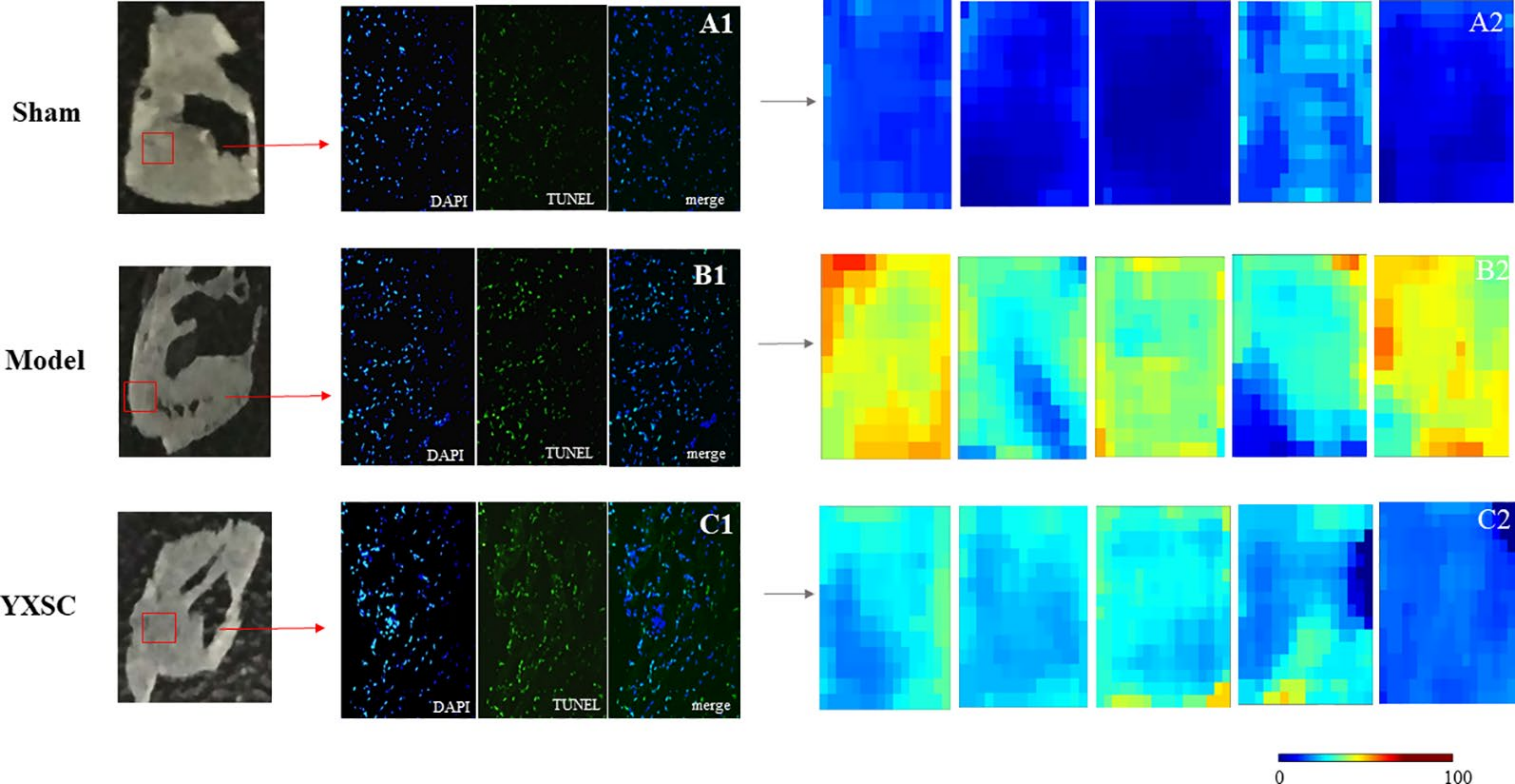
*In situ* MALDI-MS and the levels of all metabolites at the apoptotic section of the hearts from sham group, model group, and YXSC group. Microscope images for whole Tunel stained heart sections from sham group **(A1)**, model group **(B1)**, and YXSC group **(C1)** (40 X). Heat map describing the total ion intensity at the region of apoptosis on the heart section from sham group **(A2)**, model group **(B2)**, and YXSC group **(C2)**.

**Figure 5:**
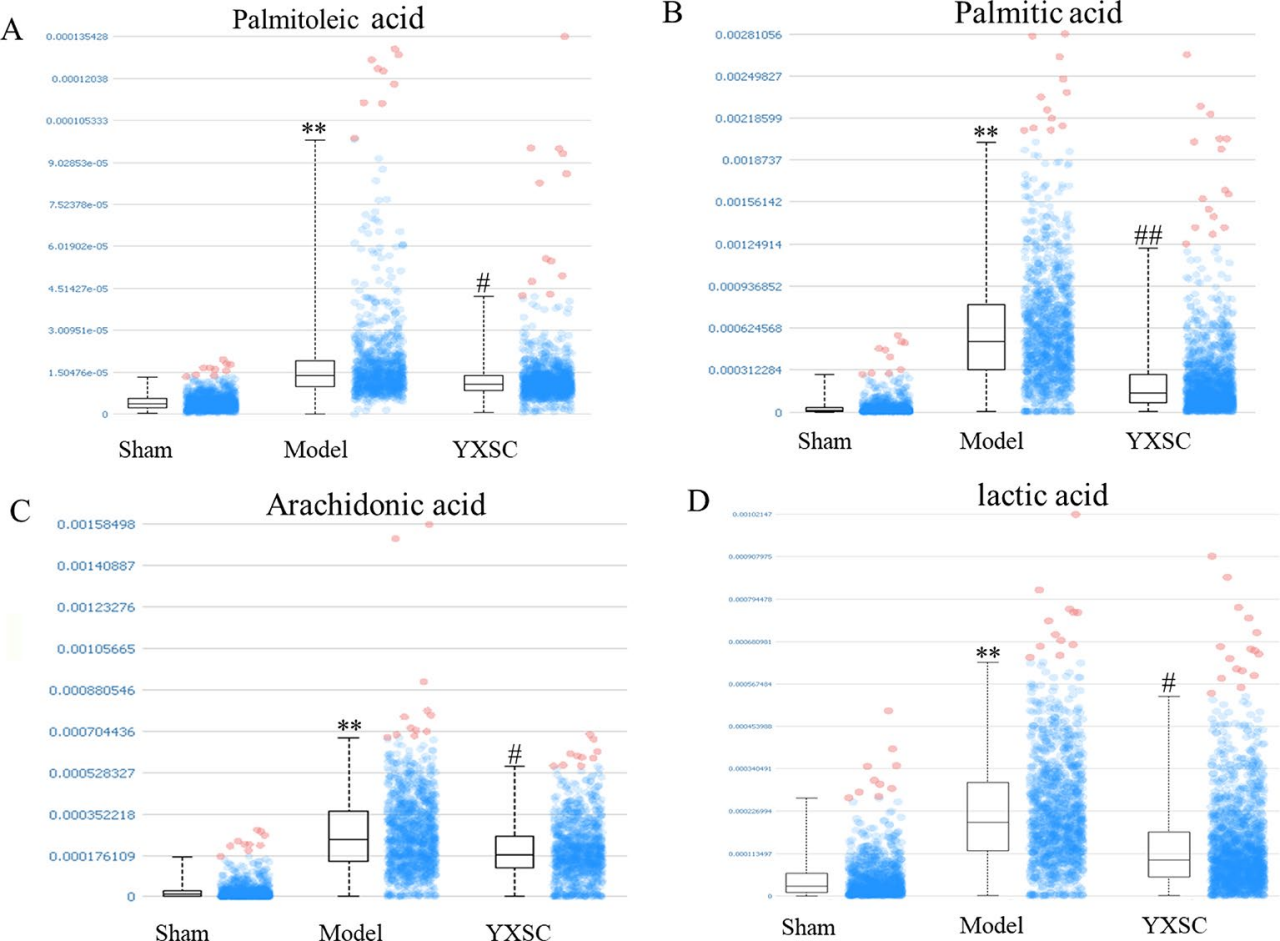
The ion intensity of palmitoleic acid, palmitic acid, arachidonic acid, and lactic acid extracted from the region of apoptosis on the heart section. Data were shown as the mean ± SD (n = 5). Asterisk mark and octothorpe mark indicate significant differences (** *p* < 0.01, sham group versus model group; ## *p* < 0.01, # *p* < 0.05, model group versus YXSC group). **(A)** (Palmitoleic acid, [M-H]^-^ m/z 253.217), **(B)** (Palmitic acid, [M-H]^-^ m/z 255.237), **(C)** (Arachidonic acid, [M-H]^-^ m/z 303.234), **(D)** (lactic acid [M-H]^-^ m/z 89.025).

*In situ* MALDI-MS ionization was applied to verify plasma metabolite markers at the apoptosis region of the heart. As shown in **Figures 4 A1, B1, and C1**, TUNEL stained images were used to locate the apoptosis region of heart induced by ischemia. Then, the parallel section from the same rat hearts (n = 5) was subjected to MALDI-MS. Imaging data were acquired in the negative ionization mode at a spatial resolution of 200 µm. As shown in **Figures 4 A2, B2, and C2**, based on total ion current, the image reflects a relative modification of all metabolite levels at the apoptosis region of the heart between the sham group, model group, and YXSC group. Furthermore, the target metabolite markers found in the plasma were extracted by exact mass search as listed in **Table 1**, using the high accuracy of m/z determined by TOF-MS. The identification was further confirmed by MS/MS fragmentation. Finally, four metabolites were selected, including palmitoleic acid, palmitic acid, arachidonic acid, and lactic acid, for verification in the heart tissue. **Figure 5** showed the ion intensity of palmitoleic acid, palmitic acid, arachidonic acid, and lactic acid at the apoptosis region of the heart in the sham group, model group, and YXSC group. It can be seen from inspection of **Figure 5** that the ion intensities of palmitoleic acid, palmitic acid, arachidonic acid, and lactic acid in the model group were all higher than those in the sham group. Furthermore, a tendency for returning to baseline values in YXSC group (*p* < 0.05) could be determined, consistent to the variation trends in the plasma.

### Correlation Network of Differential Metabolic Markers

**Figure 6:**
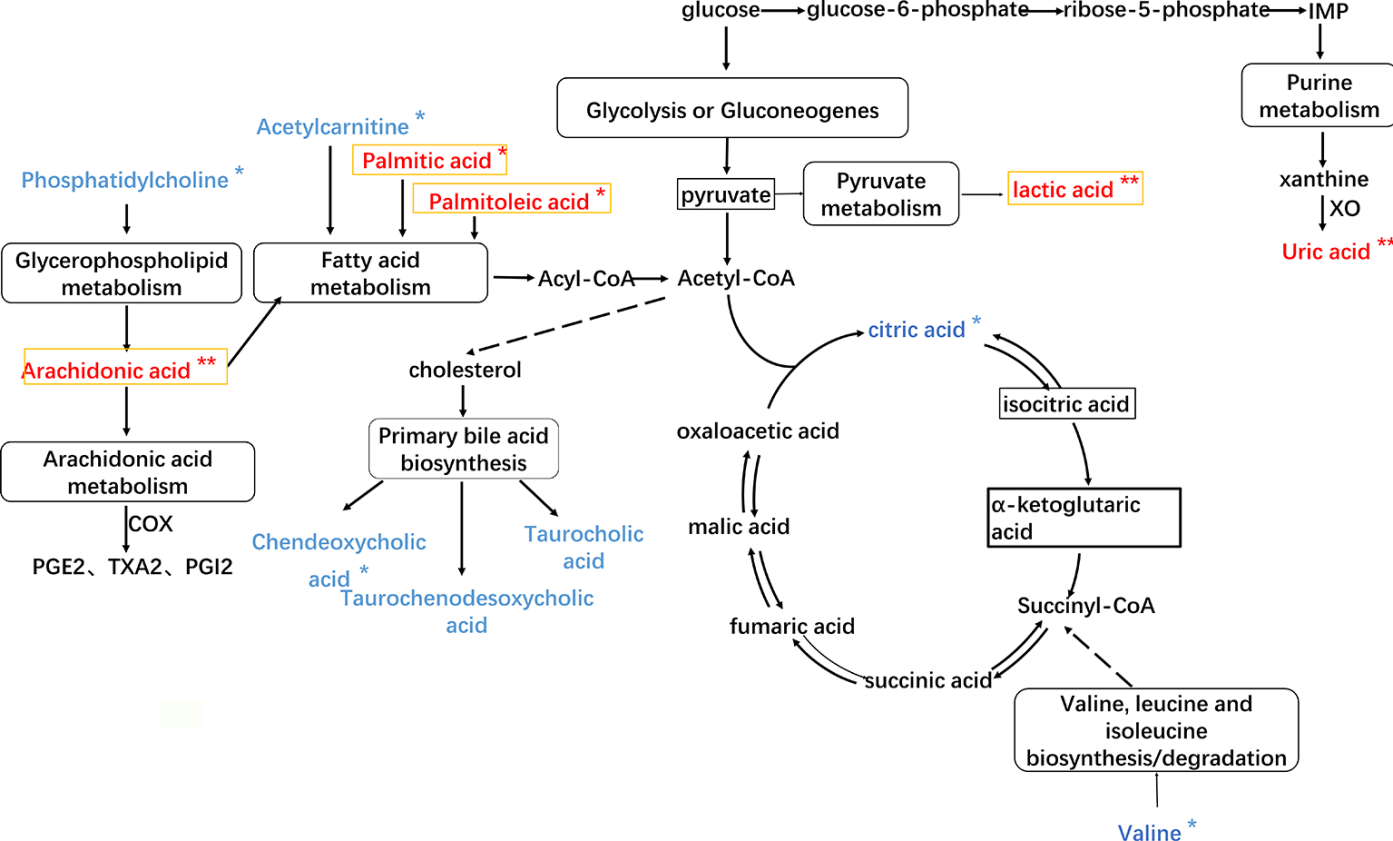
The HF imbalanced network of the metabolic markers in plasma of rat. Metabolites in red and blue represent increase and decrease changes in HF rats; metabolites in yellow rectangle represent validation by MALDI-MS. 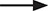 Direct interaction, 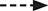 Indirect interaction, ** *p* < 0.01, *p < 0.05, YXSC group versus model group.

In order to investigate the latent relationships of the metabolic markers of HF in the plasma, a correlation network diagram was constructed based on the KEGG database. As shown in **Figure 6**, the network diagram showed the interrelationship based on the identified metabolic pathways including 12 metabolic markers. The metabolic pathways of glycerophospholipid metabolism, arachidonic acid metabolism, fatty acid metabolism, primary bile acid biosynthesis, glycolysis or gluconeogenesis, pyruvate metabolism, purine metabolism, and amino acid metabolism are related to each other *via* acetyl coenzyme A (Acetyl-CoA) and the citric acid cycle. In the network diagram shown in **Figure 5**, the metabolites in red and blue indicate changes, i.e., amount increase and decrease, in the blood of HF rats compared to the sham groups, respectively. Importantly, the asterisk mark indicates that the metabolic markers showed a tendency for returning to baseline values in the YXSC group. Moreover, metabolites in the heart tissue highlighted by a yellow rectangle were further validated by MALDI-MS.

## Discussion

To this day, the optimization of cardiac substrate metabolism to improve cardiac function and to slow the progression of HF, without causing any direct negative hemodynamic or inotropic effects, is believed to represent an attractive therapeutic approach. Although the consequences of metabolic dysfunction in HF are poorly understood, there is growing evidence to support the concept that alterations in substrate metabolism significantly contribute to contractile dysfunction and the progression of left ventricular (LV) remodeling. The metabolic markers identified in this study may offer an improved understanding of the complex pathogenesis of HF and the potential YXSC mechanism *in vivo*.

### Fatty Acid and Pyruvate Metabolism

Plasma fatty acid concentration is generally regulated by the net release of fatty acids from triglycerides in adipocytes, reflecting the net balance between triglyceride breakdown by hormone-sensitive lipase and synthesis by glycerol phosphate acyltransferase (Lopaschuk et al., 1994). Under conditions of metabolic stress, such as ischemia, diabetes, or starvation, plasma free fatty acid concentrations may reach significantly increased levels. Furthermore, elevated fatty acid levels in the plasma can result in a cardiac specific “lipotoxicity,” characterized by accumulation of neutral lipids (triglycerides) and ceramides, which are associated with apoptosis, and contractile dysfunction (Stanley et al., 2005). It has already been shown that ceramide, a critical second messenger in the regulation of signal transduction associated with apoptosis, participates in various biochemical processes including lipid peroxidation, bile acid biosynthesis, fatty acid biosynthesis, fatty acid metabolism, and lipid transport (Djoumbou Feunang et al., 2016).

Palmitic acid represents one of the most common saturated fatty acids and serves as precursor of ceramide synthesis as well as inhibitor of mitochondrial adenine nucleotide translocase. Palmitic acid is generally associated with apoptosis in the heart and has pro-apoptotic properties in cardiomyocytes (Vries et al., 1997; Sparagna et al., 2004; Miller et al., 2005). Palmitoleic acid, an unsaturated fatty acid, is positively associated with hypertension, inflammation, and cardiovascular mortality, critical risk factors for HF (Zheng et al., 1999; Warensjö et al., 2008; Mozaffarian et al., 2010; Djousse et al., 2012). In clinical study, the levels of palmitoleic acid have been shown to be significantly elevated in HF patients (Lopaschuk et al., 1994; Fosshaug et al., 2015; Ruiz et al., 2017). In the study reported herein, the levels of palmitoleic acid and palmitic acid in the plasma were found to be increased in HF rats. Furthermore, using MALDI-MS, both levels could also be detected with the same variation tendency in the heart. The results were consistent with published reports and further suggest that palmitoleic acid and palmitic acid may represent important metabolic markers for fatty acid metabolism disorder in HF.

In addition, generally appreciated is the notion that the energy required for heart function mainly results from fatty acids metabolism and carbohydrate metabolism. Myocardial ischemia has been shown to disturb the net balance of “glucose-fatty acid cycle,” resulting in a decreased mitochondrial ATP production, increased glucose anaerobic glycolysis, lactic acid accumulation, and cytotoxicity (Randle et al., 1963; Randle et al., 1964). Therefore, increasing the oxidation of glucose and lactic acid and thereby converting the energy metabolism of cardiomyocytes from fatty acids to glucose metabolism may contribute to improve cardiac function. As shown in **Figure 6**, lactic acid is produced from pyruvate *via* the enzyme lactate dehydrogenase (LDH) in the pyruvate metabolism. In this study, the plasma metabolic profiles analysis and MALDI-MS imaging of the heart both showed that the level of lactic acid was increased in HF rats. After treatment with YXSC, the levels of palmitoleic acid, palmitic acid, and lactic acid all showed a tendency for returning to baseline values in the YXSC group (*p* < 0.05). This important finding indicates that administration of YXSC may lead to the regulation of the fatty acid metabolism as well as pyruvate metabolism during HF. Additionally, L-acetylcarnitine, which reduces fatty acid oxidation, contributes to the improved recovery of the cardiac output in isolated perfused rat hearts subjected to global ischemia (Paulson et al., 1986). In this study, the level of L-acetylcarnitine was determined to be significantly reduced in the model group compared with the sham group. After treatment with YXSC, the level of L-acetylcarnitine showed a tendency for returning to baseline values in the YXSC group (*p* < 0.05).

### Phosphatidylcholines and Bile Acids Metabolism

Phosphatidylcholines (PCs), a major lipid component of membranes, play an important role in physiology and pathophysiology (Kim et al., 2015). There are reports that the imbalance of phosphatidylcholines metabolism may lead to functional impairment of the heart involving plaque development and progression associated with atherosclerotic cardiovascular disease (Meikle et al., 2014). In this study, a series of six PC species was found to decrease in the model group compared with the sham group, indicating that the degradation of PCs was activated in the model group.

Arachidonic acid, a polyunsaturated omega-6 fatty acid, represents a constituent of animal phosphatides. Arachidonic acid and its metabolites such as prostaglandins and leukotrienes are considered intracellular messengers. Arachidonic acid mediates inflammation and the functioning of several organs, including the heart either directly or upon its conversion into eicosanoids (Nixon, 2009). For example, 20-hydroxyecostearonic acid (20-HETE) metabolized from arachidonic acid contributes to platelet aggregation and vasoconstriction (Jarrar et al., 2019). Inflammation cytokines such as TNF-α and IL-1β triggered Ca2^+^ imbalance contribute to cardiac remodeling, cardiomyocyte hypertrophy, cardiomyocyte apoptosis, and often lead to detrimental effects (Schirone et al., 2017). The disturbance of arachidonic acid metabolites is often related to the development of cardiovascular diseases, such as hypertension (Bloom et al., 2017). In this study, the level of arachidonic acid increased in the model group compared with the sham group using UPLC-Q/TOF-MS and MALDI-MS.

Bile acids (BAs), the end products of cholesterol metabolism, served as mediators of cellular signals such as the regulator of lipid and energy metabolism (Claudel et al., 2005; Mayerhofer et al., 2017). As reported in the literature, BAs exhibit effects on the function of the cardiovascular system, including the formation of atherosclerotic plaque, influencing myocardial conduction and contraction by means of interaction with myocytes (Khurana et al., 2011). In this study, the levels of BAs found in the plasma, including chenodeoxycholic acid, taurochenodesoxycholic acid, and taurocholic acid, were reduced (*p* < 0.05 or VIP > 1) in model group compared with sham group, further demonstrating that the consumption of BAs might accelerate plaque aggregation.

After administration of YXSC, the levels of four PC species including PC (20:4/18:2), PC(18:2/16:0), PC(18:0/18:2), PC(22:6/18:0), arachidonic acid and taurocholic acid returned to normal levels. This result provided further evidence for the protective effects of YXSC, potentially due to suppressing the degradation of PCs and regulating of inflammatory reactions as well as the bile acid metabolism.

### Amino Acid Metabolism

Amino acids, forming the basic units of all proteins as well as energy-providing substrates, feature critical roles in cell signaling, biosynthesis, transportation, and key metabolic pathways. Recently reported literature data indicate that branched-chain amino acids (BCAAs) catabolic defects are associated with HF (Tanada et al., 2015; Sun et al., 2016). 3-Methyl-2-oxovaleric acid, formed from the incomplete breakdown of branched-chain amino acids, represents an abnormal metabolite and a neurotoxin (Yudkoff et al., 2005). In this study, the levels of valine, one of the BCAAs, as well as 3-methyl-2-oxovaleric acid found in the plasma showed both decreased and increased concentrations in the model group compared to the sham group.

Another amino acid, creatine, is critically involved in energy metabolism. In the context of HF, ATP levels were reported to decrease with mitochondrial abnormalities (Meyer et al., 1984; Starling et al., 1998; Sabbah, 2016). The creatine kinase (CK) system plays a vital role in buffering the cellular ATP and ADP concentrations through transferring high-energy phosphates from phosphocreatine (PCr) to ADP. The predominance of creatine is a consequence of maintaining ATP and PCr levels (Meyer et al., 1984; Zhang et al., 2014). A recent study based on ^1^H-NMR spectroscopy has demonstrated creatine depletion in human HF (Ingwall and Weiss, 2004). In a similar fashion, we observed significantly declined levels of creatine in the model group. These changes in creatine levels are closely associated with impaired cardiac function. Moreover, after treatment with YXSC, the levels of valine and creatine both showed apparent tendencies to return back to normal concentration levels.

## Conclusion

In this study, a metabonomic approach using an integrated UPLC-Q/TOF-MS technique and MALDI-MS was carried out to explore potential metabolic biomarkers and to increase the understanding of HF as well as to assess the potential mechanism of YXSC in an ischemia-induced HF rat model. Plasma metabolic profiles were analyzed by UPLC-Q/TOF-MS, with complementary HILIC and reversed-phase liquid chromatography. In an effort to identify more reliable potential metabolic biomarkers, common metabolic markers found at different time points of the 2nd, 4th, and 10th week after permanent occlusion were selected through multivariate data analysis. Furthermore, MALDI-MS was applied to identify the identified metabolic markers in the bloodstream at the apoptotic position of the heart tissue.

Based on echocardiographic assessment, in this rat model, HF could be observed at the fourth week after permanent occlusion. Moreover, clear separations could be detected between the sham group and model group by the loading plots of OPLS-DA at different time points after permanent occlusion. The potential markers of interest were extracted from the combining S-plots, variable VIP values (VIP > 1) and t-test (*p* < 0.05). In doing so, 21 common metabolic biomarkers over the course of the development and progression of HF after permanent occlusion could be identified that were mainly related to disturbances in the fatty acid metabolism, phosphatidylcholines metabolism, bile acids metabolism, amino acids metabolism, and pyruvate metabolism. Of the biomarkers, 16 metabolites such as palmitoleic acid, arachidonic acid, and lactic acid showed obvious changes (*p* < 0.01) and a tendency for returning to baseline values in YXSC-treated HF rats at the 10th week after permanent occlusion. Moreover, four biomarkers of palmitoleic acid palmitic acid, arachidonic acid, and lactic acid could be further validated by MALDI-MS at the apoptotic position of the coronal heart slice.

To summarize, an integrated UPLC-Q/TOF-MS technique and MALDI-MS strategy may provide a significant contribution and a potential analytical tool for understanding of the complex pathogenesis of various disease states. Moreover, the results obtained throughout this study may help develop novel strategies to explore the mechanism of HF and may also be useful for the study of potential biochemical mechanisms associated with YXSC effects.

## Data Availability Statement

The datasets analyzed in this manuscript are not publicly available. Requests to access the datasets should be directed to whw9905012@163.com.

## Ethics Statement

The animal study was reviewed and approved by the Academy of Chinese Medical Science’s Administrative Panel on Laboratory Animal Care. Written informed consent was obtained from the owners for the participation of their animals in this study.

## Author Contributions

Participated in research design: HWW, HY, GL, ST. Conducted experiments of bioanalysis: XYL, JW, XQL, HWW. Conducted experiments of HF rats: JX, FZ, LT, DL, YZ, XT, HHW. Performed data analysis: JX, FZ, ST. Wrote or contributed to the writing of the manuscript: HWW, JX.

## Funding

This study was financially supported by the National Natural Science Foundation of China (No. 81973711, No. 81573726, No.81673700) and the National Science and Technology Major Project (2014ZX09201021-009).

## Conflict of Interest

The authors declare that the research was conducted in the absence of any commercial or financial relationships that could be construed as a potential conflict of interest.
