# Supplementary material for: Integrated UPLC-Q/TOF-MS Technique and MALDI-MS to Study of the Efficacy of YiXinshu Capsules Against Heart Failure in a Rat Model
Source: Front Pharmacol. 2019 Dec 6;10:1474. doi: 10.3389/fphar.2019.01474 (PMC6910235; doi:10.3389/fphar.2019.01474)
Supplement: Supplementary file 1 [file Table_1.docx]

**Supplementary materials**

**1 Quality control of Yixinshu Capsule**

Yixinshu Capsule (YXSC), recorded in the Chinese Pharmacopoeia (Chinese Pharmacopoeia Commission, 2015), derived from a classic TCM prescription named Sheng-Mai-San, contains seven Chinese herbal medicines including *Radix Salviae Miltiorrhizae*, *Fructus Schisandra chinensis*, *Panax ginseng*, *Radix ophiopogonis*, *Astragalus membranaceus*, *RhizomaChuanxiong* and *Fructus Crataegi*.

The components of YXS Capsules are mainly comprised by the chemical substances from the involved single herbs. Therefore, the components in YXS Capsule mainly contain ginsenosides from *Panax ginseng*, astragalus saponins from *Astragalus membranaceus*, ophiopogonins and homoisoflavonoids from *Radix ophiopogonis*, lignans from *Fructus Schisandrae Chinensis*, tanshinones from *Radix Salviae Miltiorrhizae*, flavonoids from *Astragalus membranaceus* and *Fructus Crataegi*, lactones from *Rhizoma Chuanxiong*, phenolic acids from *Radix Salviae Miltiorrhizae*. In our previous studies, a total of 276 components in the YXSC were identified mainly including ginsenosides, astragalus saponins, lignans, phenolic acids and tanshinones ^[1]^.

As a Chinese Materia Medica standardized product approved by China National Medical Products Administration (NMPA), the quality control of YXSC complied with the standards in Chinese Pharmacopoeia (total content of ginsenoside Re and Rg1 were not less than 0.4 mg per capsule and salvianolic acid B was not less than 1.0 mg per capsule by HPLC)^[2]^. Furthermore, in order to comprehensively guarantee the quality of YXSC, a new HPLC method for quantitatively detecting nine compounds including protocatechuic acid, chlorogenic acid, ferulic acid , rosmarinic acid, lithospermic acid, senkyunolide I, salvianolic acid B, cryptotanshinone, tanshinone ⅡA was developed. The results indicate that the quality of YXSC used in this study is controlled and stable.

**1.2** **Materials and methods**

**1.2.1 chemicals**

An HPLC analysis was performed by Waters ACQUITY UPLC System (Waters Technologies, USA). HPLC-grade methanol and acetonitrile were supplied by Thermo Fisher Scientific Inc. (Shanghai, China). Deionized water was purchased (Wahaha, China). The reference compounds protocatechuic acid, chlorogenic acid, ferulic acid , rosmarinic acid, lithospermic acid, senkyunolide I, salvianolic acid B, cryptotanshinone, tanshinone ⅡA (purity >98% for all) were purchased from Chengdu Chroma-Biotechnology Co., Ltd. (Sichuan, China).

**1.2.2 Sample preparation**

The powder of Yixinshu Capsule content (0.5 g) was added to a 100 mL Erlenmeyer flask with 50 mL methanol, and the flask was accurately weighed. Following soaking for 0.5 h and ultrasonic extraction for 30 min, the sample mixture was weighed again and any solvent lost in the process was added after being cooled to room temperature. Subsequently, the mixture was filtered through a 0.22 μm membrane filter. Finally, 2 μL aliquots from the filtrate were subjected to HPLC analysis. Stock solutions of the 9 reference compounds of protocatechuic acid, chlorogenic acid, ferulic acid, rosmarinic acid, lithospermic acid, senkyunolide I, salvianolic acid B and cryptotanshinone, tanshinone Ⅱ - of about 0.2 mg/mL - were prepared in methanol and stored at 4°C for later analysis.

**1.2.3 Chromatographic and mass spectrometric conditions**

An HPLC analysis was performed by Waters ACQUITY UPLC System equipped with thermostat autosampler, diode array detector (DAD) and Empower 3 workstation (Waters Technologies, USA). The chromatographic column was used Waters ACQUITY HSS T3 柱(2.1 mm×10 mm, 1.8 μm). The mobile phase was composed of solvent A (0.5% formic acid-water) and solvent B (0.5% formic acid-acetonitrile) with a gradient elution (0~1 min, 3%~4%B; 1~3min, 4%~14.3%B; 3~4min, 14.3%~18.1%B; 4~7 min, 18.1%~27.9%B; 7~10.2min, 27.9%~57%B; 10.2~13min, 57%~72.5%B; 13~15min, 72.5%~100%B.). The flow rate of the mobile phase was 0.4 mL•min^-1^. The column temperature was maintained at 37°C, and the sample manager temperature was set at 4°C. The injection volume was 2 µL^-1^. The detective wavelengths were selected at 254 nm ( protocatechuic acid), 265 nm(cryptotanshinone, tanshinone ⅡA); 277 nm (senkyunolide I), 280 nm (rosmarinic acid, lithospermic acid, salvianolic acid B), and 322 nm (chlorogenic acid, ferulic acid) respectively.

**1.3 Results**

All the nine analytes were successfully separated using the developed HPLC method as shown in Figure S1. The presence of each of the nine compounds in the samples was confirmed from the retention time and UV spectra. The contents of the 9 compounds in YXSC used in this study were calculated according to their respective calibration curves. The results are shown in Table S1.

Table S1 The contents of the 9 compounds in YXSC used in this study ($\bar{x}$, n=3, mg/g)

| protocatechuic acid | chlorogenic acid | ferulic acid | rosmarinic acid | lithospermic acid | salvianolic acid B | senkyunolide I | cryptotanshinone | tanshinoneⅡA |
| --- | --- | --- | --- | --- | --- | --- | --- | --- |
| 0.2078 | 0.0951 | 0.0223 | 0.4017 | 0.6376 | 18.1777 | 0.1325 | 1.0509 | 0.6616 |


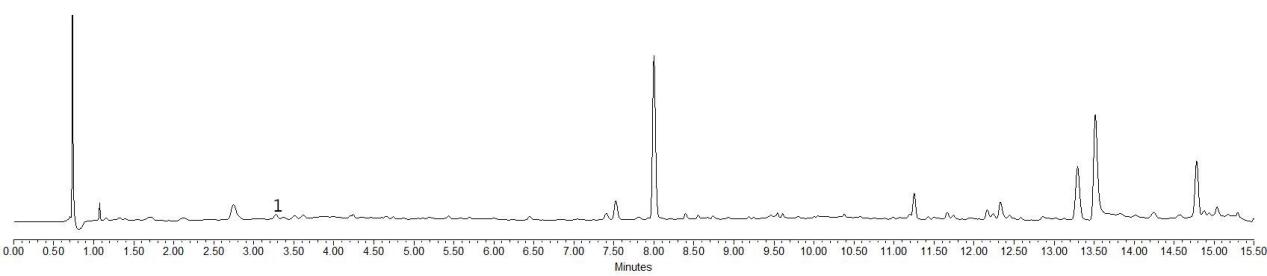


(a)


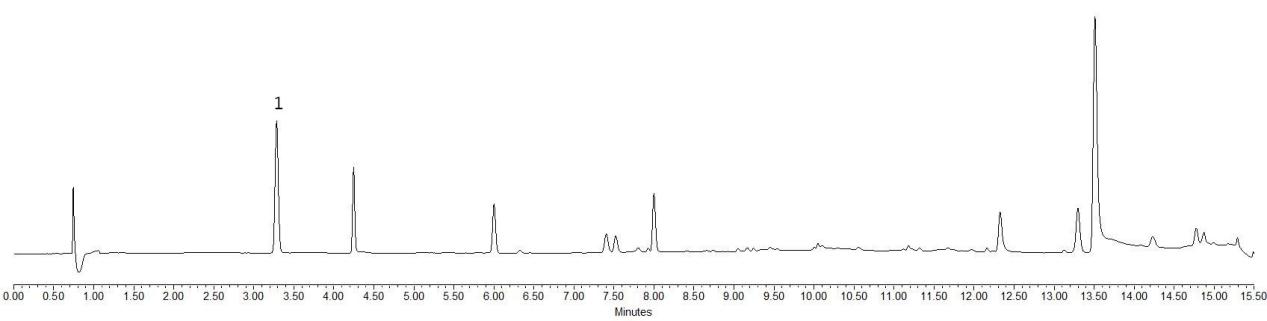


(b)


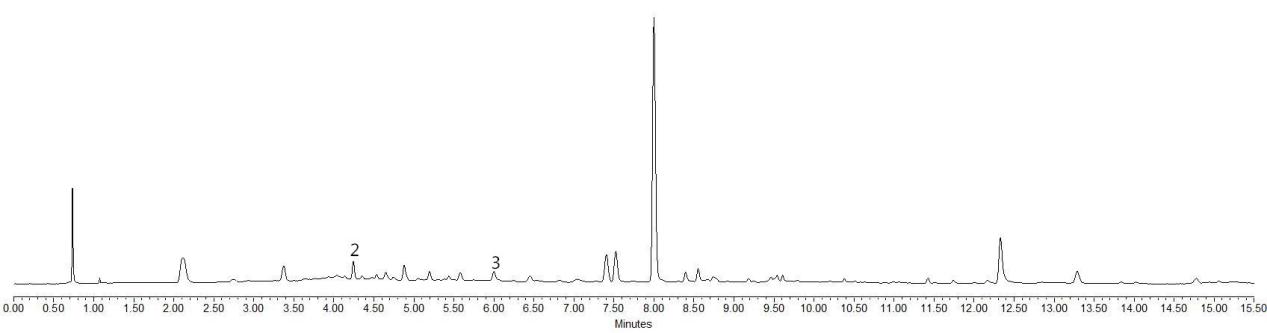


(c)


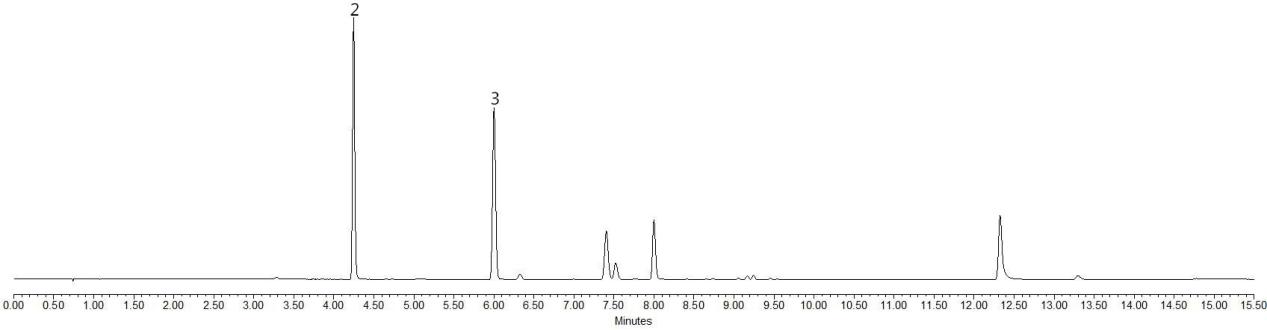


(d)


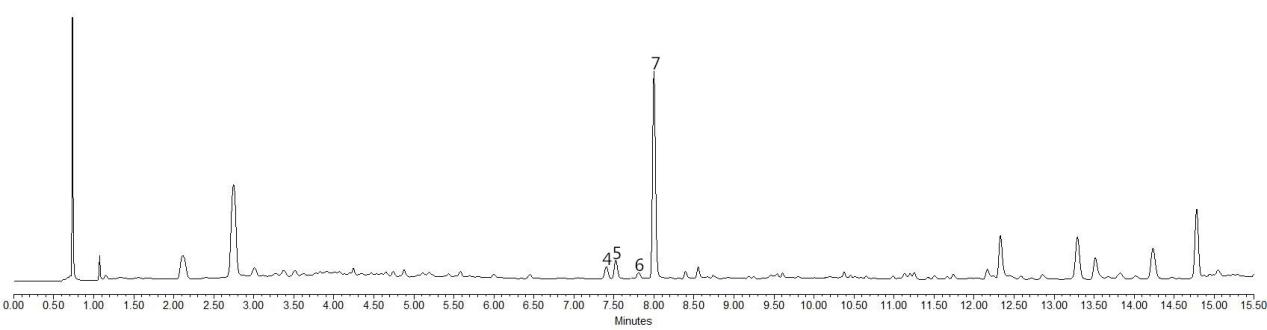


(e)


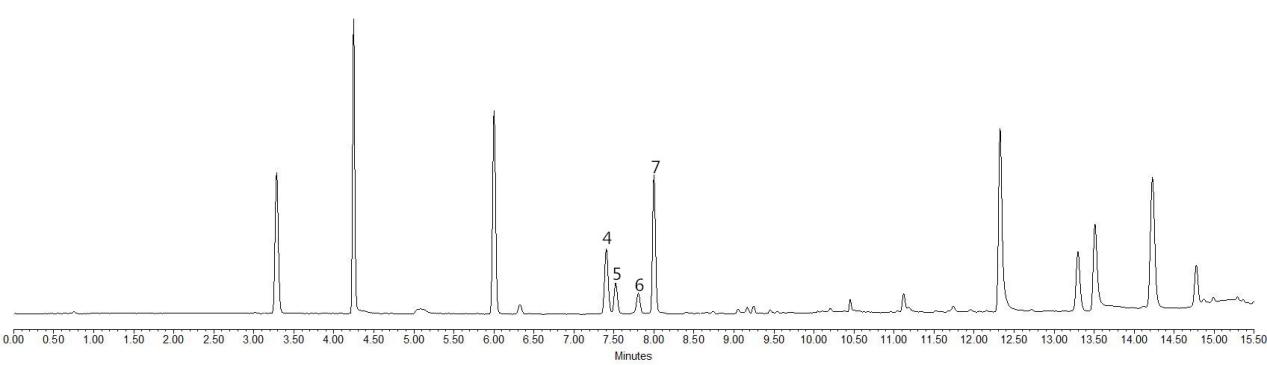


(f)


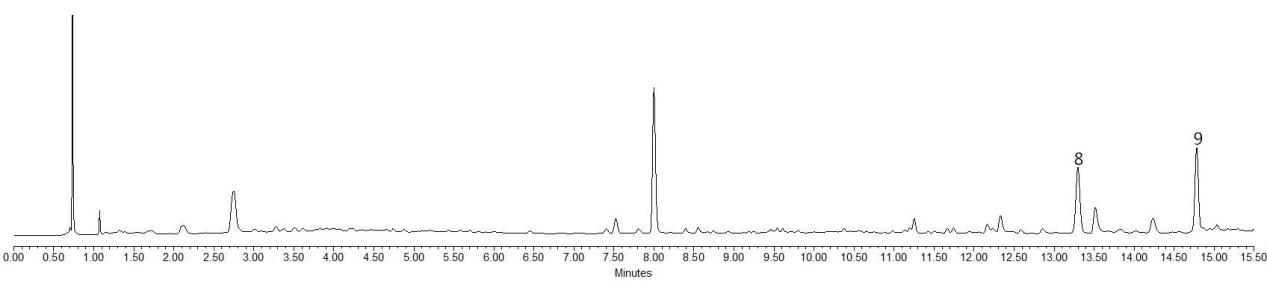


(g)


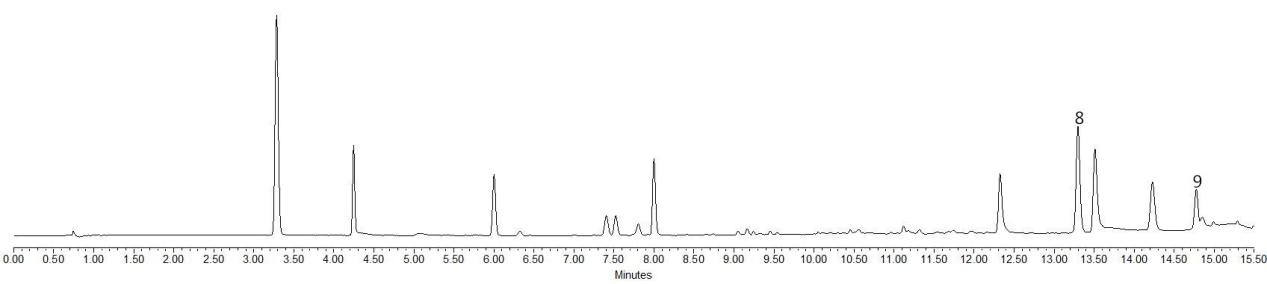


(h)

Figure S1 The chromatography of determination of 9 components in YXSC by UPLC

(a): samples at 254nm, (b):mix standards at 254nm; (c): samples at 322nm, (d): mix standards at 322nm; (e): samples at 280nm, (f): mix standards at 280nm; (g): samples at 265nm; (h): mix standards at 265nm. (1. protocatechuic acid, 2. chlorogenic acid, 3. ferulic acid, 4. rosmarinic acid, 5. lithospermic acid(lithospermic acid), 6. senkyunolide I, 7. salvianolic acid B, 8. cryptotanshinone, 9. tanshinone Ⅱ_A_)

1. protocatechuic acid 2. chlorogenic acid 3. ferulic acid

4. rosmarinic acid 5. lithospermic acid(lithospermic acid) 6. senkyunolide I

7. salvianolic acid B 8. cryptotanshinone 9. tanshinone Ⅱ_A_

Figure S2 The structures of the 9 compounds detected in YXSC

**2. The relative contents of the identified common metabolic markers between the sham group and model group at the second and four week after permanent occlusion**

Table. S2 The relative contents of the identified common metabolic markers between the sham group and model group at the second and fourth week after permanent occlusion

| No. | Name | Normalized content (×10000) at the second week after MI | |  | Normalized content (×10000) at the fourth week after MI | |
| --- | --- | --- | --- | --- | --- | --- |
|  |  | Sham | Model |  | Sham | Model |
| 1 | PC(20:4/18:2) | 481.43±12.28 | 410.47±11.44 |  | 456.78±20.20 | 301.21±40.77 |
| 2 | PC(20:4/20:4) | 350.84±6.74 | 333.11±3.04 |  | 332.56+±45.20 | 299.88±24.20 |
| 3 | PC(18:2/16:0) | 1671.24±5.35 | 1277.04±13.16 |  | 1466.45±114.21 | 1008±97.20 |
| 4 | PC(18:0/18:2) | 781.87±9.26 | 652.74±5.37 |  | 789±78.20 | 623.76±74.22 |
| 5 | PC(20:4/18:0) | 1698.31±10.56 | 1390.01±23.64 |  | 1876.34±211.20 | 1544.55±104.20 |
| 6 | PC(22:6/18:0) | 500.69±3.97 | 473.60±5.27 |  | 451.88±68.22 | 389.98±78.20 |
| 7 | 3-Methyl-2-oxovaleric acid | 65.95±5.85 | 71.41±8.94 |  | 55.75±12.21 | 77.56±24.20 |
| 8 | Palmitoleic acid | 42.05±11.72 | 46.08±8.87 |  | 46.89±10.78 | 87.11±7.45 |
| 9 | Palmitic acid | 664.31±18.72 | 885.42±13.69 |  | 613.24±89.61 | 667.87±55.61 |
| 10 | Chenodeoxycholic acid | 17.26±9.72 | 15.90±6.07 |  | 21.55±9.81 | 15.32±4.61 |
| 11 | Taurochenodesoxycholic acid | 4.76±13.76 | 4.05±2.88 |  | 6.77±0.61 | 2.32±0.31 |
| 12 | Arachidonic acid | 365.20±16.96 | 434.72±10.09 |  | 198.34±25.18 | 250.65±40.22 |
| 13 | Taurocholic acid | 78.93±9.51 | 38.56±4.48 |  | 60.34±7.08 | 30.36±5.28 |
| 14 | Lactic acid | 17.99±8.22 | 20.69±16.69 |  | 19.67±3.48 | 32.14±5.48 |
| 15 | Citric acid | 155.97±14.06 | 136.46±8.06 |  | 110.22±17.23 | 68.67±10.08 |
| 16 | Uric acid | 9.91±9.07 | 13.21±12.69 |  | 13.22±1.06 | 26.67±2.12 |
| 17 | Valine | 103.27±22.46 | 79.15±29.81 |  | 87.56±17.45 | 36.48±10.78 |
| 18 | Edetic acid | 168.24±17.03 | 134.57±13.40 |  | 198.67±25.66 | 145.78±88.68 |
| 19 | Creatine | 57.35±4.73 | 46.29±15.51 |  | 36.78±5.67 | 16.76±7.45 |
| 20 | L-acetylcarnitine | 213.17±8.23 | 186.16±10.40 |  | 186.67±44.32 | 138.36±87.65 |
| 21 | N1-Methyl-2-pyridone-5-carboxamide | 9.45±8.27 | 7.77±3.34 |  | 15.67±2.08 | 10.21±7.00 |

P<0.05 or VIP >1 for all the values between the sham group and model group

**References**

[1] Hong-ping Wang, Chang Chen, Yan Liu, Hong-jun Yang, Hong-wei Wu, Hong-bin Xiao. Identification of the chemical constituents of Chinese medicine Yi-Xin-Shu capsule by molecular feature orientated precursor ion selection and tandem mass spectrometry structure elucidation. J. Sep. Sci. 2015, 38, 3687–3695

[2] Pharmacopoeia of China, Committee for the pharmacopoeia of China, Part I; China Medical Science and Technology Press, Beijing, China, 2015; pp. 1400–1401.
